# GTF2I Mutation in Thymomas: Independence From Racial-Ethnic Backgrounds. An Indian/German Comparative Study

**DOI:** 10.3389/pore.2021.1609858

**Published:** 2021-08-23

**Authors:** Deepali Jain, Prerna Guleria, Varsha Singh, Rajinder Parshad, Sunil Kumar, Timo Gaiser, Katrin S. Kurz, German Ott, Stefan Porubsky, Gerhard Preissler, Christian G. Sauer, Sebastian Schölch, Philipp Ströbel, Thomas Hielscher, Alexander Marx, Zoran V. Popovic

**Affiliations:** ^1^ Department of Pathology, All India Institute of Medical Sciences, New Delhi, India; ^2^ Department of Surgery, All India Institute of Medical Sciences, New Delhi, India; ^3^ Surgical Oncology, All India Institute of Medical Sciences, New Delhi, India; ^4^ Institute of Pathology, University Medical Centre Mannheim, University of Heidelberg, Mannheim, Germany; ^5^ Dr. Margarete Fischer-Bosch Institute for Clinical Pharmacology, Stuttgart, Germany; ^6^ Department of Clinical Pathology, Robert-Bosch-Krankenhaus, Stuttgart, Germany; ^7^ Institute of Pathology, University Hospital, Johannes-Gutenberg University Mainz, Mainz, Germany; ^8^ Department of Thoracic Surgery, Schillerhöhe Clinics, Robert-Bosch-Krankenhaus, Gerlingen, Germany; ^9^ Department of Surgery, University Medical Centre Mannheim, University of Heidelberg, Mannheim, Germany; ^10^ Junior Clinical Cooperation Unit Translational Surgical Oncology, German Cancer Research Center (DKFZ), Heidelberg, Germany; ^11^ Institute of Pathology, University Medical Center Göttingen, University of Göttingen, Göttingen, Germany; ^12^ Department of Biostatistics, German Cancer Research Center (DKFZ), Heidelberg, Germany

**Keywords:** epidemiology, thymoma, GTF2I mutation, myasthenia gravis, racial-ethnic factors

## Abstract

Thymomas are the most frequent adult mediastinal cancers. Their etiology is unknown and their pathogenesis poorly understood. Racial, ethnic and environmental factors influence tumorigenesis in many cancers, but their role in thymomas remains unclear to date. In this study that included pretreatment thymoma cases from India and Germany (*n* = 37 and *n* = 77, respectively) we compared i) the prevalence of the thymoma-specific chromosome 7 c.74146970T > A mutation of the *GTF2I* gene in type A and AB thymomas; ii) epidemiological features; and iii) the frequency of myasthenia gravis (MG). Due to a known predominance of GTF2I mutation in A and AB histotypes, we included only a marginal number of type B thymomas as a control group in both cohorts. While the distribution of histological types between the cohorts was similar (*p* = 0.1622), Indian patients were strikingly younger (*p* < 0.0001; median age 50 vs. 65 years) and showed significantly lower tumour stage (Masaoka-Koga stage I) at primary diagnosis (*p* = 0.0005) than the German patients. In patients with known MG status (*n* = 17 in Indian and *n* = 25 in German cohort), a clear trend towards more frequent MG was observed in the Indian group (*p* = 0.0504; 48 vs. 82%). The prevalence of the *GTF2I* mutation (analysed in *n* = 34 Indian and *n* = 77 German patients) was identical in the two cohorts. We conclude that racial-ethnic and environmental factors do not significantly influence the most common molecular feature of thymomas but may have an impact on the timing of clinical presentation.

## Background

Thymic epithelial tumours (TET)are the most common tumours arising in the anterior mediastinum in adults, comprising mainly thymomas and the rarer and more aggressive thymic carcinomas (1). The focus here is on thymomas that are subdivided into type A, AB, B1, B2, and B3 and rare other subtypes according to histological criteria of the WHO classification. Studies on the genomic landscape of TETs are limited. Nevertheless, the few studies conducted have demonstrated a genetic variability between the various histologic types thereby indicating that the subtypes are not only morphologically but also molecularly distinct (2-4). So much so that some authors have even identified molecular subtypes of TETs and have associated them with clinical phenotypes and outcomes (5, 6). Among various other genomic alterations, the most common one is the point mutation in chromosome 7 c.74146970T > A of the *general transcription factor II-I* (*GTF2I*) gene that has been identified to be specific to thymic epithelial tumours, predominantly in type A and AB thymomas and associated with a more favourable prognosis(7, 8). Its prevalence in type B1-B3 thymomas and thymic carcinomas is much lower, with decreasing frequency as the histology progresses to a more aggressive subtype (7). Since the mutation has not been widely studied with respect to different racial and ethnic backgrounds, we aimed to identify its prevalence in two populations from different regions of the world and identify any geographical variability if present in the molecular profile of thymomas. With this objective in mind, we carried out a retrospective analysis, to assess the frequency of *GTF2I* mutation in Indian and German thymomas (mainly type A and AB) and to correlate it with clinical features.

## Materials and Methods

The study was carried out as collaboration between the departments of pathology at the University Medical Centre Mannheim, Heidelberg University, Mannheim, Germany and the All India Institute of Medical Sciences, New Delhi. A cohort of Indian (*n* = 37) and German (*n* = 77) thymomas enriched for A and AB histotypes with a primary goal to address the GTF2I mutation frequency was retrieved from the departmental archives. Pretreatment surgical resections were performed in the period 2008–2018 and included no recurrent tumor or metastatic tissue. The clinical details of these cases were obtained from case files. Hematoxylin-eosin stained slides were reviewed by two experts in the field (AM and DJ) in terms of histotype (1)and Masaoka-Koga stage (9) that allows for a more detailed description of early invasion than the TNM system (see *Discussion*). Primers for Exon 15 of the *GTF2I* gene were designed using National Center for Biotechnology Information (NCBI) and Primer 3 (v.0.4.0) software.

### DNA Isolation and Quantification

Genomic DNA was isolated from formalin-fixed, paraffin embedded (FFPE) samples using 50-µm-thick sections of the tumour according to the manufacture’s protocol using ReliaPrep gDNA Tissue Miniprep system (cat. No. A2050, Promega, United States). Only paraffin blocks where cancer occupied >50% of the section were selected for DNA extraction. The isolated DNA was subjected to qualitative and quantitative assessment *via* spectrophotometry-based absorbance studies using Nanodrop, Biodrop Resolution, Cambridge, United Kingdom. Polymerase Chain Reaction (PCR) of GTF gene (exon 15) was carried out in a 25 µL total volume, containing 80 ng genomic DNA, 10 µM respective primers and using GoTaq colorless master mix (cat. No. M7142, Promega, United States).

### Polymerase Chain Reaction and Sanger Sequencing

The PCR program was set as: HotStarsTaq activation at 95°C for 5 min, followed by 40 cycles, each having denaturation at 95°C for 30 s, annealing at 55°C for 1 min and extension at 72°C for 45 s, and the final extension was 72°C for 10 min. The quality of the PCR product was assessed using electrophoresis by resolving a part of the PCR amplified product (3 µl) on 1.5% agarose gel for all considered samples. Clear band of expected size was observed in all samples which corresponded to the size of the desired amplicons. The PCR product was then cleaned up manually using polyethylene glycol (PEG) purification methods. The PEG purified PCR product was used for sequencing PCR with a total reaction volume of 10 µl. The sequencing PCR was set as: initial denaturation at 95°C, 5 min, followed by 35 cycles, each having denaturation at 95°C for 10 s, annealing at 55°C for 5 s and extension at 60°C for 4 min. The sequencing PCR product was cleaned up using 125 µM EDTA, 100% ethanol, and 3 M sodium citrate. Post-clean up, DNA was reconstituted in HiDi and incubated at 95°C for 5 min for denaturation and immediately put on ice to cool the PCR product. After this snap chilling, the samples were proceeded for sequencing. The sequencing was done using both forward and reverse primers (GTF2I_F: 5′-ATC​CCG​TAC​CCT​CTT​TTC​C-3′, GTF2I_R: 5′-AGA​CAA​GAG​TTC​AAC​AGG-3′) for greater accuracy and the results were analysed using SeqMan II software (DNASTAR).

### Immunohistochemistry

For immunohistochemical staining of representative thymoma cases, paraffin embedded tissue was cut to 1 µm sections. The staining was performed using an automated slide preparation system (Benchmark Ultra, Roche Tissue Diagnostics, Ventana, Tucson, AZ, United States), a Ventana OptiView DAB IHC Detection Kit (Ventana, Tucson, AZ, United States)and commercially available monoclonal antibodies for CD20 (Clone L26, DCS, Hamburg, Germany), TdT (Clone SEN28, Leica Biosystems, Buffalo Grove, IL, USA) and CK19 (Clone RCK108, Agilent, Santa Clara, CA. United States).

### Statistical Analyses

Primary endpoint of this study was the *GTF2I*mutational status. Subgroup analyses and analysis of MG status were exploratory. Associations between clinico-pathological and molecular factors and geographical origin were assessed with Fisher’s exact test for categorical factors and with Wilcoxon test for age. Age-adjusted effects of clinical parameters were tested with the likelihood-ratio test of logistic regression models for origin between a model with predictors clinical parameter and age and a model with predictor age only. Heterogeneity of association between origin and mutational status across subgroups was analysed with the likelihood-ratio test between a model with origin and subgroup factor as predictors and a model including interaction term of both predictors. *p*-values below 0.05 were considered significant. Statistical software R 4.0 was used to carry out analyses.

## Results

### Cohort Data and Histological Distribution

A total of 138 resection specimens were retrieved of which 37 were Indian cases and 101 were German. Among these, 114 cases were thymomas consisting of 23 (20.2%) type A thymomas, 52 (45.6%) type AB, 17 atypical A/AB (14.9%) and 22 (19.3%) type B1-B3 thymomas. German thymoma cases (*n* = 77) included 33 female (42,9%) and 44 male (57,1%) patients in the age range 31–85 years (median age 65 years). Masaoka-Koga stage I was reported in 16/77 cases (20,8%), stage II in 38 cases (49,4%), stage III in 9 cases (11,7%) and stage IV in 14 cases (18,2%). The German cohort included 15 (19,5%) type A thymomas, 31 (40,3%) type AB, 15 atypical A/AB (19,5%) and 16 (20,8%) type B1-B3 thymomas. The remaining 24 German cases comprised 18 thymic carcinomas, 2 cases of sclerosing mediastinitis, 2 metaplastic thymomas, 1 thymolipoma and 1 thymic hyperplasia with lymphoepithelial sialadenitis (LESA)-like features (10).

**FIGURE 1:**
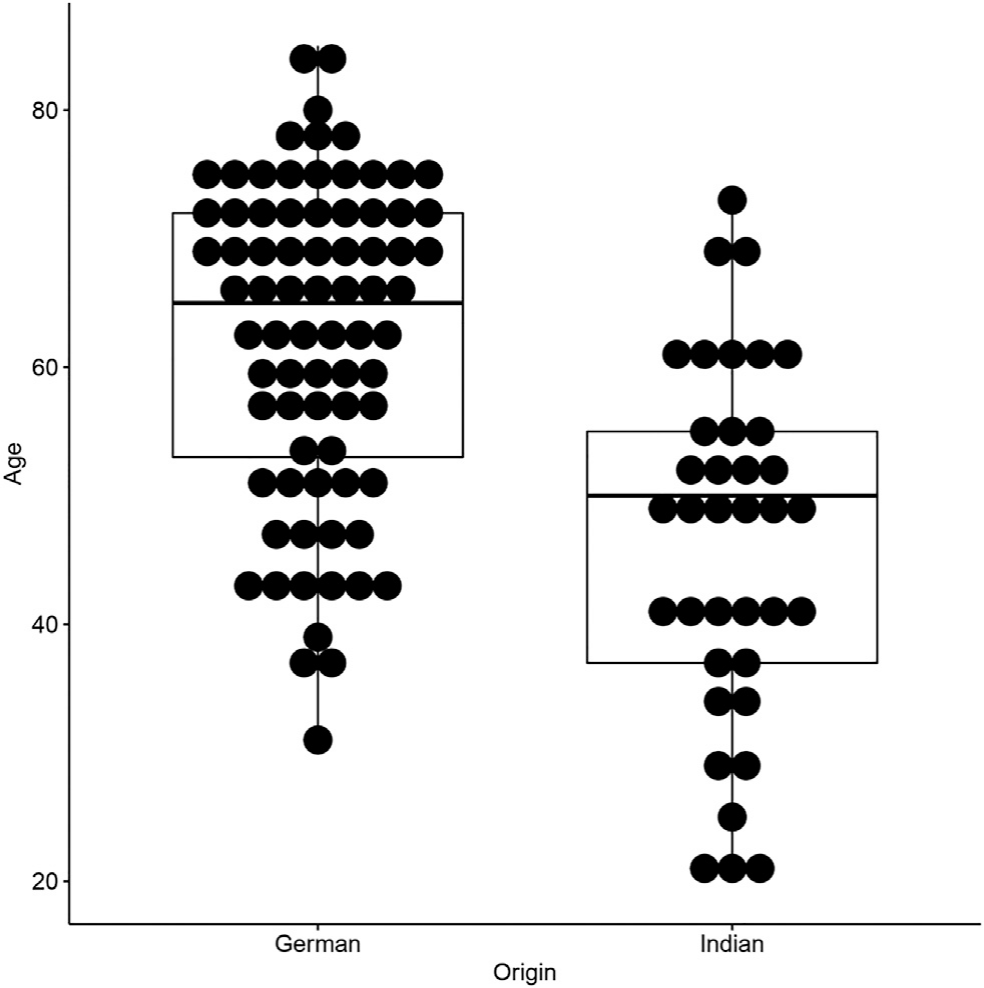
Age-distribution plot of Indian and German patients included in this study.

All the 37 Indian cases included in this study were thymomas [8 type A (21.6%), 21 type AB (56.8%), 2 atypical A/AB (5.4%)and 6 type B1-B3 thymomas (16.2%)]. Among the 37 Indian cases, there was an equal gender distribution (19 cases or 51.4% were females). The age range in the Indian group was 20–73 years with a median age of 50 years; hence, the Indian cohort was significantly younger than the German counterpart (*p* < 0.0001, Table 1; Figure 1). Notably, even after excluding type B thymomas, this striking age difference remained in separate analyses of histotypes A and AB (*p* = 0.00097 and *p* = 0.0068, respectively; Supplementary Table S1). In both cohorts there were no significant differences between the age distribution of patients with and without MG. Masaoka-Koga stage distribution was as follows: 24 (64.9%) stage I, 9 (24.3%) stage II and 4 cases (10.8%) were in stage III.

**TABLE 1:** Contingency table of clinico-pathological factors between origins (for gender, MG status, Masaoka-Koga stage, GTF2I mutation status, histological type and age).

	Level	German	Indian	*p*	Age-adjusted *p*
N	—	77	37	—	—
Gender (%)	Female	33 (42.9)	19 (51.4)	0.4270	0.20
	Male	44 (57.1)	18 (48.6)	—	—
MG Status (%)^a^	No	13 (52.0)	3 (17.6)	0.0504	0.13
	MG	12 (48.0)	14 (82.4)	—	—
Masaoka-Koga stage (%)	I	16 (20.8)	24 (64.9)	<0.0001	0.0003
	II	38 (49.4)	9 (24.3)	—	—
	III/IV	23 (29.9)	4 (10.8)	—	—
Mutation status (%)^b^	WT	28 (36.4)	12 (35.3)	1.00	0.26
	L404H	49 (63.6)	22 (64.7)	—	—
Histological type (%)	A	15 (19.5)	8 (21.6)	0.1622	0.048
	AB	31 (40.3)	21 (56.8)	—	—
	atypical A/AB	15 (19.5)	2 (5.4)	—	—
	B	16 (20.8)	6 (16.2)	—	—
Age [median (IQR)]	—	65.00 (53.00, 72.00)	50.00 (37.00, 55.00)	<0.0001	—

MG—myasthenia gravis; IQR—interquartile Range.

a MG status was known in 25 German and 17 Indian cases.

b GTF2I Mutation was analysed in 77 German and 34 Indian cases.

**FIGURE 2:**
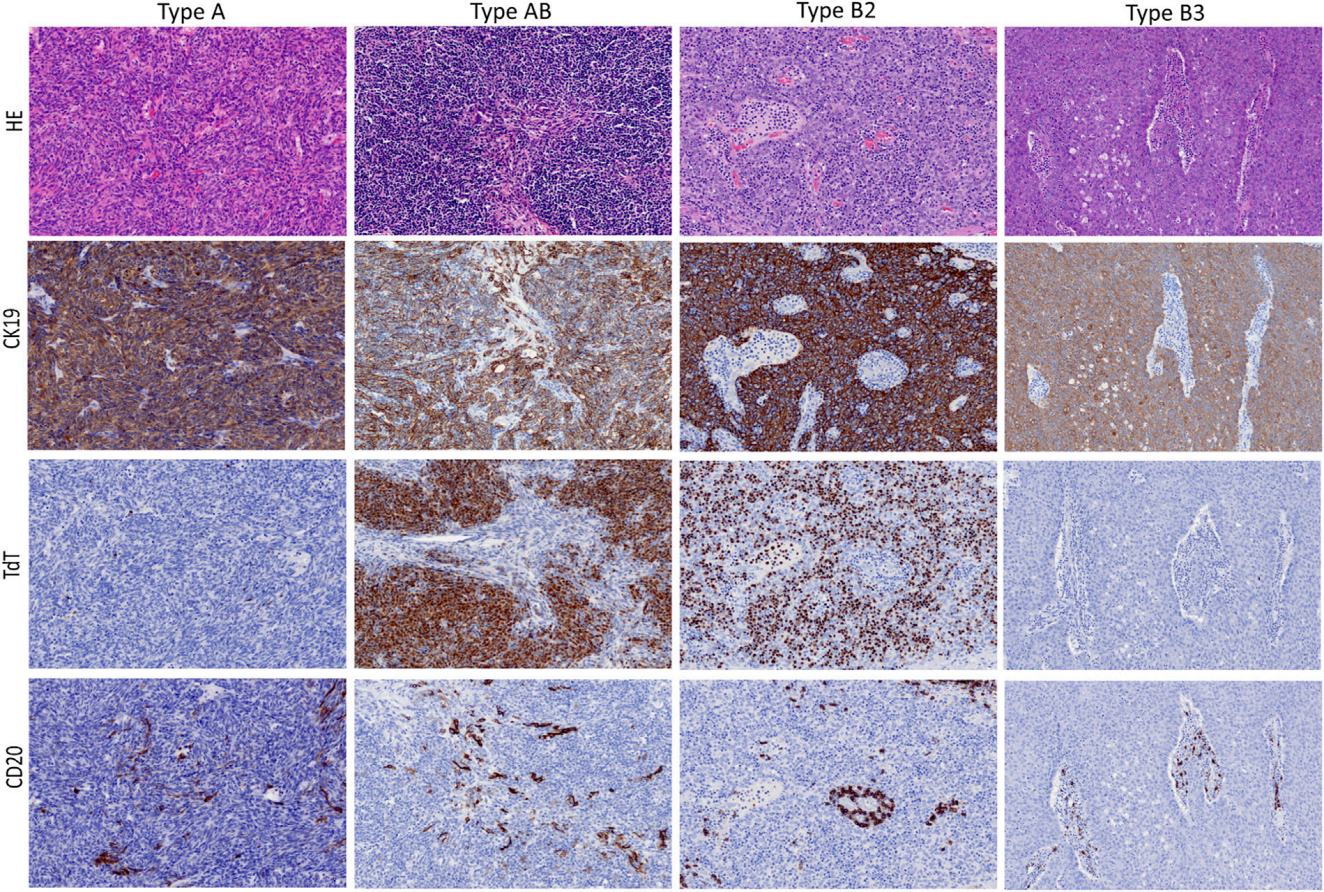
Typical morphological presentation of WHO thymoma histotypes included in this study (upper lane; HE, 20x objective) and corresponding immunohistochemical expression pattern (mid and low lanes; 20x objective).

Within defined WHO thymoma histotypes, we could not observe morphologic variations that could be assigned as specific to the Indian or German cohort, as confirmed by two experts in the field (AM and DJ). Representative histological and immunohistological features of thymoma cases included in this study are illustrated in Figure 2.

Also, no significant difference regarding the distribution of thymoma histotypes was evident (*p* = 0.16; Table 1). In age-adjusted analysis, there was a difference (*p* = 0.048), because age and histotype are strongly associated. On the other hand, we could detect a highly significant difference with respect to disease stage at clinical presentation, showing a clear majority of Indian patients at Masaoka-Koga Stage I (64.9% of all Indian thymoma cases) in comparison to only 20.8% of German patients at this early stage (*p* < 0.0001; Table 1). In our histotype A- and AB-dominated cohorts, this difference was most prominent for type AB (*p* = 0.006) followed by type A thymomas (*p* = 0.03; Tables 2 and 3).

**TABLE 2:** Contingency table of clinico-pathological factors between origins (for histological type A).

	Level	German	Indian	*p*
N	—	15	8	—
Gender (%)	Female	6 (40.0)	6 (75.0)	0.193
	Male	9 (60.0)	2 (25.0)	—
MG Status (%)	No	2 (100.0)	1 (14.3)	0.083
	MG	0 (0.0)	6 (85.7)	—
Masaoka-Koga stage (%)	I	3 (20.0)	6 (75.0)	0.032
	II	6 (40.0)	2 (25.0)	—
	III/IV	6 (40.0)	0 (0.0)	—
Mutation status (%)	WT	6 (40.0)	1 (12.5)	0.345
	L404H	9 (60.0)	7 (87.5)	—
Age [median (IQR)]	—	70.00 (66.00, 73.50)	50.00 (41.50, 56.75)	0.001

MG—myasthenia gravis; IQR—interquartile Range.

**TABLE 3:** Contingency table of clinico-pathological factors between origins (for histological type AB).

	Level	German	Indian	*p*
N	—	31	21	—
Gender (%)	Female	13 (41.9)	11 (52.4)	0.573
	Male	18 (58.1)	10 (47.6)	—
MG Status (%)	No	7 (36.8)	0 (0.0)	0.272
	MG	12 (63.2)	5 (100.0)	
Masaoka-Koga stage (%)	I	11 (35.5)	15 (71.4)	0.006
	II	19 (61.3)	4 (9.0)	—
	III/IV	1 (3.2)	2 (9.5)	—
Mutation status (%)	WT	2 (6.5)	4 (22.2)	0.175
	L404H	29 (93.5)	14 (77.8)	—
Age [median (IQR)]	—	59.00 (50.00, 70.50)	50.00 (41.00, 55.00)	0.007

MG—myasthenia gravis; IQR—interquartile Range.

### Association With Myasthenia Gravis

In the German cohort, follow up information on myasthenia gravis status was available in 25/77 thymomas (of which 19 were type AB, 2 type A and 4 atypical A/AB) and 12 patients had myasthenia gravis (48% of patients with available information on myasthenia status). All tumours arising in myasthenia-positive German patients were diagnosed as type AB thymoma in Masaoka-Koga stage I or II (TNM: pT1a stage I).

*Myasthenic status was known in 17 Indian patients, of these 14 had MG* (82.3%) of which 6 (42.9%) had type A, 5 (35.7%) type AB thymomas and 3 cases (21.4%) type B thymomas. The single myasthenic patient with an atypical type A thymoma had recurrence of disease, and another patient with a “conventional” type A thymoma died due to myasthenic crisis. High risk features seen in the atypical A thymoma were increased atypia of the epithelial cells along with frequent mitotic activity and areas of necrosis.

**FIGURE 3:**
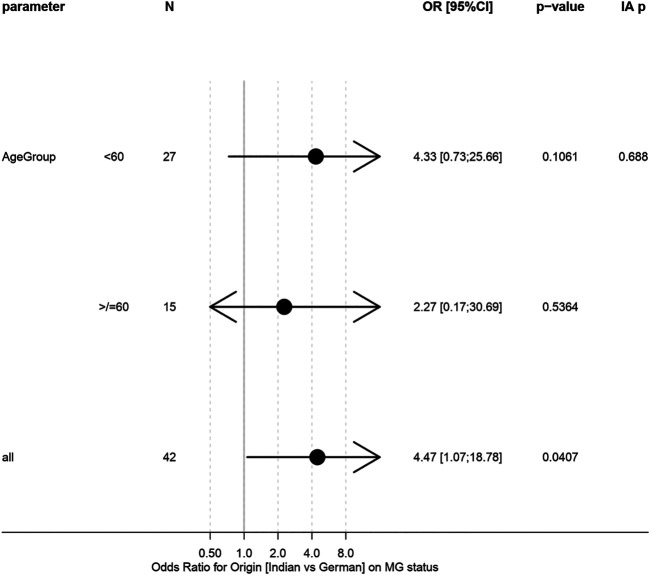
Forest plot of logistic regression models for association between origin and MG status in age subgroups. “IA *p*” gives test on interaction/h`eterogeneity between subgroups.

Hence, a clear tendency towards significantly more frequent MG could be seen in the Indian cohort (82 vs. 48%; *p* = 0.0504; Table 1). When accounting for age differences between cohorts, no significant effect remained (*p* = 0.13; Table 1). No significant difference in association between geographical origin and MG status could be observed between age groups (*p* = 0.69; Figure 3). Intriguingly, all German patients with MG had thymomas of AB histotype, compared with only 35.7% of Indian patients (Tables 4 and 5).

**TABLE 4:** Contingency table of clinico-pathological factors between origins (for patients with MG).

	Level	German	Indian	*p*
N	—	12	14	—
Gender (%)	Female	5 (41.7)	9 (64.3)	0.431
	Male	7 (58.3)	5 (35.7)	—
Histological type (%)	A	0 (0.0)	6 (42.9)	0.001
	AB	12 (100.0)	5 (35.7)	—
	atypical A/AB	0 (0.0)	0 (0.0)	—
	B	0 (0.0)	3 (21.4)	—
Masaoka-Koga stage (%)	I	3 (25.0)	10 (71.4)	0.047
	II	9 (75.0)	4 (28.6)	—
	III/IV	0 (0.0)	0 (0.0)	—
Mutation status (%)	WT	1 (8.3)	4 (28.6)	0.330
	L404H	11 (91.7)	10 (71.4)	—
Age [median (IQR)]	—	55.00 (48.50, 71.25)	48.00 (35.50, 51.50)	0.025

MG—myasthenia gravis; IQR—interquartile Range.

**TABLE 5:** Contingency table of clinico-pathological factors between origins (for MG-free patients).

	Level	German	Indian	*p*
n	—	13	3	—
Gender (%)	Female	4 (30.8)	1 (33.3)	1.000
	Male	9 (69.2)	2 (66.7)	—
Histological type (%)	A	2 (15.4)	1 (33.3)	0.095
	AB	7 (53.8)	0 (0.0)	—
	atypical A/AB	4 (30.8)	1 (33.3)	—
	B	0 (0.0)	1 (33.3)	—
Masaoka-Koga stage (%)	I	4 (30.8)	1 (33.3)	0.750
	II	4 (30.8)	0 (0.0)	—
	III/IV	5 (38.5)	2 (66.7)	—
Mutation status (%)	WT	3 (23.1)	2 (66.7)	0.214
	L404H	10 (76.9)	1 (33.3)	—
Age [median (IQR)]	—	62.00 (58.00, 74.00)	42.00 (32.00, 55.00)	0.157

MG—myasthenia gravis; IQR—interquartile Range.

The analysis of clinico-pathological factors between MG and non-MG patients (pooled German and Indian groups) showed significant differences in Masaoka-Koga stage between the two groups. Patients with MG presented at earlier stage (100% MG versus 56.2% non-MG pateints at Masaoka-Koga stage I or II; *p* = 0.0013; Table 6).

**TABLE 6:** Contingency table of clinico-pathological factors between patients without and with MG in both cohorts.

	Level	No MG	MG	*p*
N	—	16	26	—
Gender (%)	Female	5 (31.2)	14 (53.8)	0.2077
	Male	11 (68.8)	12 (46.2)	—
Origin (%)	German	13 (81.2)	12 (46.2)	0.0540
	Indian	3 (18.8)	14 (53.8)	—
Masaoka-Koga stage (%)	I	5 (31.2)	13 (50.0)	0.0013
	II	4 (25.0)	13 (50.0)	—
	III/IV	7 (43.8)	0 (0.0)	—
Mutation status (%)	WT	5 (31.2)	5 (19.2)	0.4649
	L404H	11 (68.8)	21 (80.8)	—
Histological type (%)	A	3 (18.8)	6 (23.1)	0.0251
	AB	7 (43.8)	17 (65.4)	—
	atypical A/AB	5 (31.2)	0 (0.0)	—
	B	1 (6.2)	3 (11.5)	—
Age [median (IQR)]	—	60.50 (56.00, 69.50)	50.00 (42.25, 60.50)	0.0370

MG—myasthenia gravis; IQR—interquartile Range.

### GTF2I Mutation Status

**FIGURE 4:**
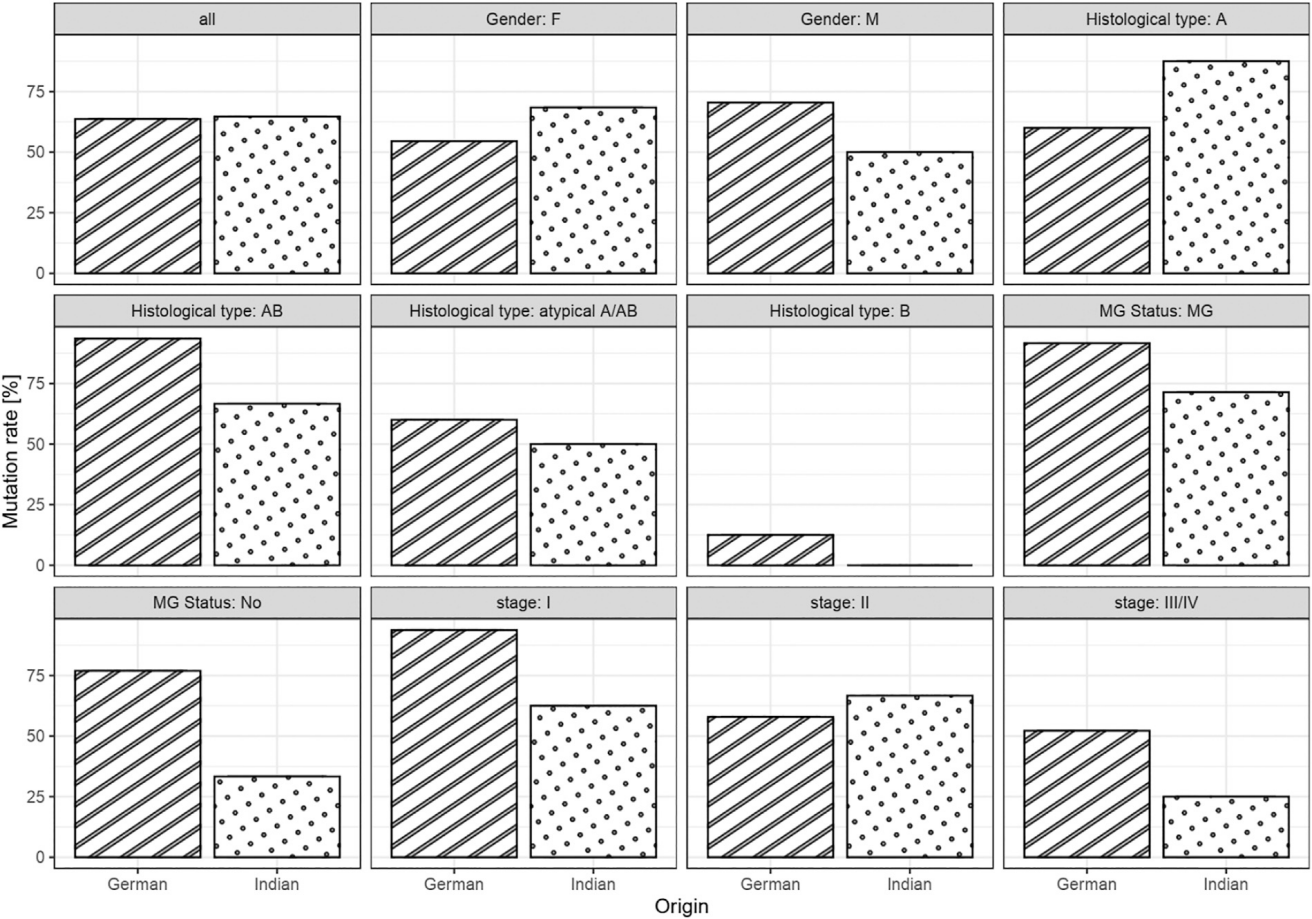
Bar chart of mutation rates between origin in the full cohorts and in subgroups.

Out of the 114 thymomas, 3 Indian cases were excluded from mutational studies since the extracted DNA was of insufficient quality. Overall, mutation rates were similar in both cohorts (64 vs. 65%, *p* = 1.00, age-adjusted *p* = 0.26; Table 1). As expected, in general there was a strong association between histotypes A and AB and GTF2I mutation (*p* < 0.0001; Table 7). Out of a total of 89 type A/AB thymomas (including atypical forms), 71 (79.8%) were positive for the *GTF2I* mutation, including 78.6% (22/28) Indian and 80% (49/61) German cases. Overall, 20.2% (18/89) of types A/AB were negative for the mutation. Among the type B thymomas, overall prevalence of the mutation was 9.1% (2/22) of which all Indian B histotypes (*n* = 6) were negative, whereas 2 of 16 (12.5%) German B thymomas were positive, hence without significant difference (*p* > 0.05). In 25 German cases with known myasthenia gravis status, the *GTF2I* mutation was evident in 11/12 patients with myasthenia gravis (91,7%) and 10/13 myasthenia-free patients (76,9%). Out of 14 Indian cases with MG, 10 (71.4%) showed the *GTF2I* mutation; 1/3 patients (33.3%) without myasthenia harboured the mutation. There was no significant association of the *GTF2I* mutational status with the prevalence of MG overall and in both cohorts. No mutation was found in non-thymomatous samples, including 18 thymic carcinomas. Association between origin and mutational status was not significantly different between clinical subgroups (Figure 4).

**TABLE 7:** Contingency table of clinico-pathological factors between patients without and with GTF2I mutation in both cohorts.

	Level	WT	L404H	*p*
N	—	40	71	—
Gender (%)	Female	19 (47.5)	31 (43.7)	0.8427
	Male	21 (52.5)	40 (56.3)	—
Origin (%)	German	28 (70.0)	49 (69.0)	1.00
	Indian	12 (30.0)	22 (31.0)	—
Masaoka-Koga stage (%)	I	8 (20.0)	30 (42.3)	0.0434
	II	19 (47.5)	28 (39.4)	—
	III/IV	13 (32.5)	13 (18.3)	—
MG Status (%)	No	5 (50.0)	11 (34.4)	0.4649
	MG	5 (50.0)	21 (65.6)	—
Histological type (%)	A	7 (17.5)	16 (22.5)	<0.0001
	AB	6 (15.0)	43 (60.6)	—
	atypical A/AB	7 (17.5)	10 (14.1)	—
	B	20 (50.0)	2 (2.8)	—
Age [median (IQR)]	—	58.00 (41.75, 73.00)	59.00 (50.00, 69.00)	0.7238

MG—myasthenia gravis; IQR—interquartile Range.

## Discussion

The potential impact of racial-ethnic, and environmental factors on the pathogenesis of thymoma has rarely been addressed. A study of the International Thymic Malignancy Interest Group (ITMIG) with over 4,000 thymomas from Asia, Europe and the Americas reported geographic differences for the prevalence of WHO thymoma histotypes between continents (11). That study did not include patients from India, i.e., a population with distinct heterogeneous germ line differences (12) and environmental features, including specific dietary habits (13). Thus, the present study is the first to address such differences through the comparison of Indian and German thymoma patients with regard to the prevalence of the thymoma-specific *GTF2I* mutation, clinical manifestation, paraneoplastic myasthenia gravis and histology. In the Indian cohort, we observed a significantly younger age at the time point of surgery, a higher prevalence of thymoma-associated MG, and a lower Masaoka-Koga (but not TNM-based) tumour stage. By contrast, gender and WHO histotype distribution, *GTF2I* mutation rates, and absent association between the presence of MG and *GTF2I* mutational status were similar in both cohorts.

As to the higher proportion of MG (+) cases among the Indian thymomas compared to the German and a previous world-wide cohorts (5, 6), referral bias needs to be taken into consideration, since the 82% MG positivity rate in the Indian cohort is much higher than any other percentage reported previously in larger series (14, 15). Furthermore, striking differences of MG frequencies in cohorts from different institutions in the same country were reported in meta-analyses and thought to reflect referral bias (14). Referral bias also could have a bearing on the lower tumour stage observed in the Indian cohort, since MG (+) thymomas may be detected earlier than MG (−) thymomas through myasthenic rather than local symptoms that are typical of larger thymomas in MG (−) patients (16). Indeed, also in our study, the different nature of the Indian and German institues should be taken into consideration: while Indian patients come from an institute that is super-specialized in neurosciences, the major focus of the German referral center is thymic pathology. This fact may contribute to the striking difference in MG prevalence between the two groups and should be appreciated as one of the limitations of the study. Therefore, we are reluctant to attribute the lower tumour stage in Indian patients to racial-ethnic differences. By contrast, the median age difference of 15 years between Indian and German thymoma patients (50 versus 65 years) is intriguing, since such a young age, for a cohort that is dominated by type A and AB thymomas is among the lowest reported to date (14, 15). However, a comparably young age was found in another Asian cohort from Korea (17). This observation needs validation in a larger and MG-unbiased cohort of Indian thymoma patients. If confirmed, a younger manifestation age may hint to an increased risk for thymoma and thymoma-associated MG development in Indians. In fact, the proportion of thymoma patients among MG patients was found to be higher in some Asian countries compared to Northern European countries (18-20).

Since gender and WHO histotype distribution as well as *GTF2I* mutation rates were similar in the Indian and German cohort and in line with a previous world-wide study (6), racial-ethnic, environmental and lifestyle factors appear not to have a major impact on the molecular mechanism(s) driving the development of thymomas. A similar conclusion was recently drawn in relation to thymic squamous cell carcinomas in which age-related rather than racial-ethnic and environmental factors appear to be related to oncogenesis (6, 21).

Our study has some limitations: First, different sample size of the two cohorts together with the overall low number of patients with known MG status in this retrospective analysis preclude the drawing of definite conclusions. Second, the very high prevalence of MG patients in the Indian cohort largely precludes the detection of any differences between Indian and German non-MG patients. Third, since the total mutational burden of thymomas is extremely low (6), the prevalence of the only common and unique mutation, *GTF2I*, was the sole readout to study molecular differences between Indian and German thymoma patients. Therefore, it will be interesting to see, whether multiomics analyses can still identify molecular thymoma features that segregate with racial-ethnic and environmental differences. Fourth, since the *GTF2I* mutation is largely restricted to type A and AB thymomas, the two patients cohorts were equally and strongly skewed towards an over-representation of these two histologies and are thus not representative regarding the histotype distribution (22). Therefore, future comparative studies between racially and ethnically disparate groups should comprise type B thymomas and thymic carcinomas as well. Finally, we had no information on the patients’ specific racial-ethnic and dietary settings, precluding a distinction between potential genetic and environmental reasons for the observed differences. These caveats underline the necessity of comprehensive molecular and epidemiological studies of larger and more representative cohorts of Indian and German thymoma patients to identify genetic and environmental risk factors for the development of thymomas and their complications.

In summary, comparing rather unique cohorts of Indian and German thymoma patients, we could not identify significant differences regarding WHO histological types, gender and *GTF2I* mutation status, implying that there are no differences in molecular pathogenesis of thymoma between the two countries. Nonetheless, the significantly younger age of Indian thymoma patients is a first hint that genetic or environmental factors could contribute to the timing and overall risk of thymoma development.

## Data Availability

The datasets presented in this study can be found in online repositories. The names of the repository/repositories and accession number(s) can be found below: https://www.ncbi.nlm.nih.gov/genbank/, BankIt2452830 GTF2I MZ015743.

## References

[1] Travis WD Brambilla E Nicholson AG Yatabe Y Austin JHM Beasley MB The 2015 World Health Organization Classification of Lung Tumors. J Thorac Oncol (2015) 10(9):1243–60. 10.1097/jto.0000000000000630 26291008

[2] Badve S Goswami C Gökmen–Polar Y Nelson RP Henley J Miller N Molecular Analysis of Thymoma. PLoS One (2012) 7(8):e42669. 10.1371/journal.pone.0042669 22912720 PMC3418289

[3] Enkner F Pichlhöfer B Zaharie AT Krunic M Holper TM Janik S Molecular Profiling of Thymoma and Thymic Carcinoma: Genetic Differences and Potential Novel Therapeutic Targets. Pathol Oncol Res (2017) 23(3):551–64. 10.1007/s12253-016-0144-8 27844328 PMC5487866

[4] Girard N Shen R Guo T Zakowski MF Heguy A Riely GJ Comprehensive Genomic Analysis Reveals Clinically Relevant Molecular Distinctions between Thymic Carcinomas and Thymomas. Clin Cancer Res (2009) 15(22):6790–9. 10.1158/1078-0432.ccr-09-0644 19861435 PMC2783876

[5] Lee H-S Jang H-J Shah R Yoon D Hamaji M Wald O Genomic Analysis of Thymic Epithelial Tumors Identifies Novel Subtypes Associated with Distinct Clinical Features. Clin Cancer Res (2017) 23(16):4855–64. 10.1158/1078-0432.ccr-17-0066 28400429 PMC5559309

[6] Radovich M Pickering CR Felau I Ha G Zhang H Jo H The Integrated Genomic Landscape of Thymic Epithelial Tumors. Cancer Cell (2018) 33(2):244–e10. 10.1016/j.ccell.2018.01.003 29438696 PMC5994906

[7] Feng Y Lei Y Wu X Huang Y Rao H Zhang Y GTF2I Mutation Frequently Occurs in More Indolent Thymic Epithelial Tumors and Predicts Better Prognosis. Lung Cancer (2017) 110:48–52. 10.1016/j.lungcan.2017.05.020 28676218

[8] Petrini I Meltzer PS Kim I-K Lucchi M Park K-S Fontanini G A Specific Missense Mutation in GTF2I Occurs at High Frequency in Thymic Epithelial Tumors. Nat Genet (2014) 46(8):844–9. 10.1038/ng.3016 24974848 PMC5705185

[9] Detterbeck FC Nicholson AG Kondo K Van Schil P Moran C. The Masaoka-Koga Stage Classification for Thymic Malignancies: Clarification and Definition of Terms. J Thorac Oncol (2011) 6(7 Suppl. 3):S1710–6. 10.1097/JTO.0b013e31821e8cff 21847052

[10] Porubsky S Popovic ZV Badve S Banz Y Berezowska S Borchert D Thymic Hyperplasia with Lymphoepithelial Sialadenitis (LESA)-Like Features: Strong Association with Lymphomas and Non-myasthenic Autoimmune Diseases. Cancers (Basel) (2021) 13(2). 10.3390/cancers13020315 PMC7830871 33467055

[11] Weis C-A Yao X Deng Y Detterbeck FC Marino M Nicholson AG The Impact of Thymoma Histotype on Prognosis in a Worldwide Database. J Thorac Oncol (2015) 10(2):367–72. 10.1097/jto.0000000000000393 25616178 PMC4318643

[12] Reich D Thangaraj K Patterson N Price AL Singh L. Reconstructing Indian Population History. Nature (2009) 461(7263):489–94. 10.1038/nature08365 19779445 PMC2842210

[13] Bhupathiraju SN Guasch-Ferré M Gadgil MD Newgard CB Bain JR Muehlbauer MJ Dietary Patterns Among Asian Indians Living in the United States Have Distinct Metabolomic Profiles that Are Associated with Cardiometabolic Risk. J Nutr (2018) 148(7):1150–9. 10.1093/jn/nxy074 29893901 PMC6251517

[14] Detterbeck FC. Clinical Value of the WHO Classification System of Thymoma. Ann Thorac Surg (2006) 81(6):2328–34. 10.1016/j.athoracsur.2005.11.067 16731193

[15] Ruffini E Guerrera F Brunelli A Passani S Pellicano D Thomas P Report from the European Society of Thoracic Surgeons Prospective Thymic Database 2017: a Powerful Resource for a Collaborative Global Effort to Manage Thymic Tumours. Eur J Cardiothorac Surg (2019) 55(4):601–9. 10.1093/ejcts/ezy448 30649256

[16] Ströbel P Bauer A Puppe B Kraushaar T Krein A Toyka K Tumor Recurrence and Survival in Patients Treated for Thymomas and Thymic Squamous Cell Carcinomas: a Retrospective Analysis. Jco (2004) 22(8):1501–9. 10.1200/jco.2004.10.113 15084623

[17] Kim DJ Yang WI Choi SS Kim KD Chung KY. Prognostic and Clinical Relevance of the World Health Organization Schema for the Classification of Thymic Epithelial Tumors. Chest (2005) 127(3):755–61. 10.1378/chest.127.3.755 15764754

[18] Boldingh MI Maniaol A Brunborg C Dekker L Lipka A Niks EH Prevalence and Clinical Aspects of Immigrants with Myasthenia Gravis in Northern Europe. Muscle Nerve (2017) 55(6):819–27. 10.1002/mus.25408 27641227

[19] Huang X Liu WB Men LN Feng HY Li Y Luo CM Clinical Features of Myasthenia Gravis in Southern China: a Retrospective Review of 2,154 Cases over 22 Years. Neurol Sci (2013) 34(6):911–7. 10.1007/s10072-012-1157-z 22806326

[20] Murai H Yamashita N Watanabe M Nomura Y Motomura M Yoshikawa H Characteristics of Myasthenia Gravis According to Onset-Age: Japanese Nationwide Survey. J Neurol Sci (2011) 305(1-2):97–102. 10.1016/j.jns.2011.03.004 21440910

[21] Yamada Y Simon-Keller K Belharazem-Vitacolonnna D Bohnenberger H Kriegsmann M Kriegsmann K A Tuft Cell-like Signature Is Highly Prevalent in Thymic Squamous Cell Carcinoma and Delineates New Molecular Subsets Among the Major Lung Cancer Histotypes. J Thorac Oncol (2021). 10.1016/j.jtho.2021.02.008 33609752

[22] Guleria P Parshad R Malik PS Ray R Pandey RM Jain D. Histotyping of Indian Thymomas: A Clinicopathologic Study from north India. Indian J Med Res (2019) 150(2):153–60. 10.4103/ijmr.IJMR_530_18 31670270 PMC6829775

