# Supplementary figures and images for: GTF2I Mutation in Thymomas: Independence From Racial-Ethnic Backgrounds. An Indian/German Comparative Study
Source: Pathol Oncol Res. 2021 Aug 23;27:1609858. doi: 10.3389/pore.2021.1609858 (PMC8419886; doi:10.3389/pore.2021.1609858)

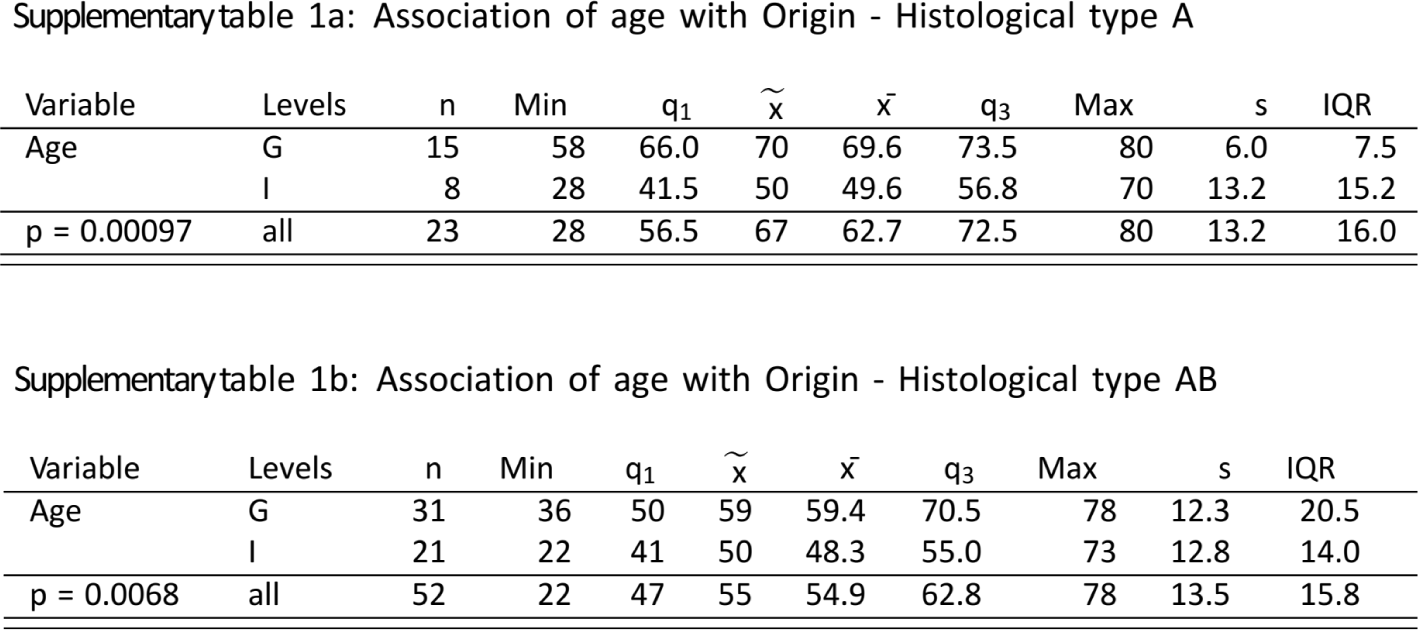

Supplement: Supplementary file 1 [file Image1.TIF]
